# Curcumae Rhizoma: A botanical drug against infectious diseases

**DOI:** 10.3389/fphar.2022.1015098

**Published:** 2023-01-10

**Authors:** Ying-Qi Wu, Tong Tong

**Affiliations:** Department of Pharmacy, The First Affiliated Hospital of USTC, Division of Life Sciences and Medicine, University of Science and Technology of China, Hefei, Anhui, China

**Keywords:** curcumin, zedoary turmeric oil, infectious diseases, antibacterial effect, antiviral effect, COVID-19, Curcumae Rhizoma

## Abstract

Curcumae Rhizoma is the dry rhizome coming from Curcuma longa L. which grow widely in tropical south and southwest Asia. It has been used to treat conditions such as dermatoses, infections, stress, and depression. Moreover, in China, Curcumae Rhizoma and its active constituents have been made into different pharmaceutical preparations. Growing evidence suggests that these preparations can exert antioxidant, anti-inflammatory, and anti-cancer effects, which may play crucial roles in the treatment of various diseases, including cancer, infectious-, autoimmune-, neurological-, and cardiovascular diseases, as well as diabetes. The anti-infective effect of Curcumae Rhizoma has become a popular field of research around the world, including for the treatment of COVID-19, influenza virus, hepatitis B virus, human immunodeficiency virus, and human papilloma virus, among others. In this paper, the basic characteristics of Curcumae Rhizoma and its active constituents are briefly introduced, and we also give an overview on their applications and mechanisms in infectious diseases.

## 1 Introduction

According to Chinese Pharmacopoeia, Curcuma longa L. has different species: Curcuma phaeocaulis Valeton, Curcuma kwangsiensis S. G. Lee, and C. F. Liang, Curcuma wenyujin Y. H. Chen and C. Ling. Curcumae Rhizoma is the dry rhizome coming from the three above. Curcumae Rhizoma commonly known as “zedoary” or “turmeric” in English and “ezhu” or “yujin” in Chinese, they are similar in source but different in medicinal parts. It is extensively cultivated in tropical and subtropical regions and used in food as a spice or ingredient in South-East Asia (Serpa Guerra et al., 2020). It was first mentioned in the theory of Medicinal Properties in China 1,400 years ago (Guo et al., 2022). Modern pharmacological research has shown that Curcumae Rhizoma has diverse pharmacological effects, including, but not limited to, antineoplastic, antibacterial, antiviral, antihyperlipidemic, anti-thrombus, and anti-liver fibrosis (Aggarwal and Harikumar, 2009; Akaberi et al., 2021).

Curcumae Rhizoma has been formulated into different traditional Chinese medicine decoctions, such as Sanleng Ezhu Decoction (Tang et al., 2021). Further, it has also been made into related pharmaceutical preparations, such as zedoary turmeric oil (ZTO) injection, antiviral oral liquid, and ZTO vaginal suppository, among others. However, because of its poor water solubility and upper gastrointestinal irritation, novel preparations are in constant development. Examples include, solid lipid nanoparticles and nanostructured lipid carriers, sustained-release microspheres, *β*-cyclodextrin, and inclusion complexes (Zhao et al., 2010; Li et al., 2017).

Currently, these preparations are widely and safely used in diseases caused by infection with microorganisms (Soleimani et al., 2018). Indeed, due to synergistic multi-component and multi-target effects, the pharmacological effects of Curcumae Rhizoma cannot be fully explained. Therefore, to further explore the role of Curcumae Rhizoma and its possible clinical applications, we searched relevant articles to systematically summarize the biological functions of these compounds and elucidate its mechanisms of action against microbial infectious diseases. The objective of this paper is to provide a reference for scientists to carry out further research on Curcumae Rhizoma.

## 2 Active constituents

Curcumae Rhizoma generally contain many components: including curcuminoid and ZTO, among others (e.g., sugars, sterols, fatty acids, alkaloids, resins, lignans, peptides, trace elements, and flavonoids) (Yuan et al., 2018). The main active components of Curcumae Rhizoma are ZTO and the non-volatile curcuminoids (Table 1).

**TABLE 1:** Main active constituents of Curcumae Rhizoma.

Component	Main chemical compound	Character	Composition
Zedoary turmeric oil	Sesquiterpenoids, monoterpenoids, diterpenoids	Volatile oils	Curcumol, germacrone, curdione, *etc.*
Curcuminoids^a^	Polyphenolic derivatives (diphenylheptane hydrocarbons, amyl hydrocarbons)	Yellow-orange powder	Curcumin (77%), demethoxycurcumin (17%) and bisdemethoxycurcumin (6%)

^a^ Curcuminoids include cyclocurcumin, but it has poor biological activity.

### 2.1 Zedoary turmeric oil

ZTO is the volatile oil extracted from Curcumae Rhizoma which is light yellow oil with special aroma (Jankasem et al., 2013; Yang et al., 2022). It was included in the Chinese Pharmacopoeia in 1977. Currently, the commonly extraction methods are steam distillation, Soxhlet extraction, microwave-assisted extraction, supercritical CO_2_ extraction and ultrasound-assisted extraction (Cai et al., 2022).

ZTO is one of the main chemical constituents in Curcumae Rhizoma and belongs to the terpenoid family, which include sesquiterpenoids, monoterpenoids, and diterpenoids, among which sesquiterpenoids are the most abundant (Li et al., 2022). Dried Curcumae Rhizoma typically contains 1.5%–5% volatile oils. Generally, the ZTO of Curcumae Rhizoma species possesses a wide variety of pharmacological properties, especially antimicrobial and antiviral properties (Dosoky and Setzer, 2018).

**FIGURE 1:**
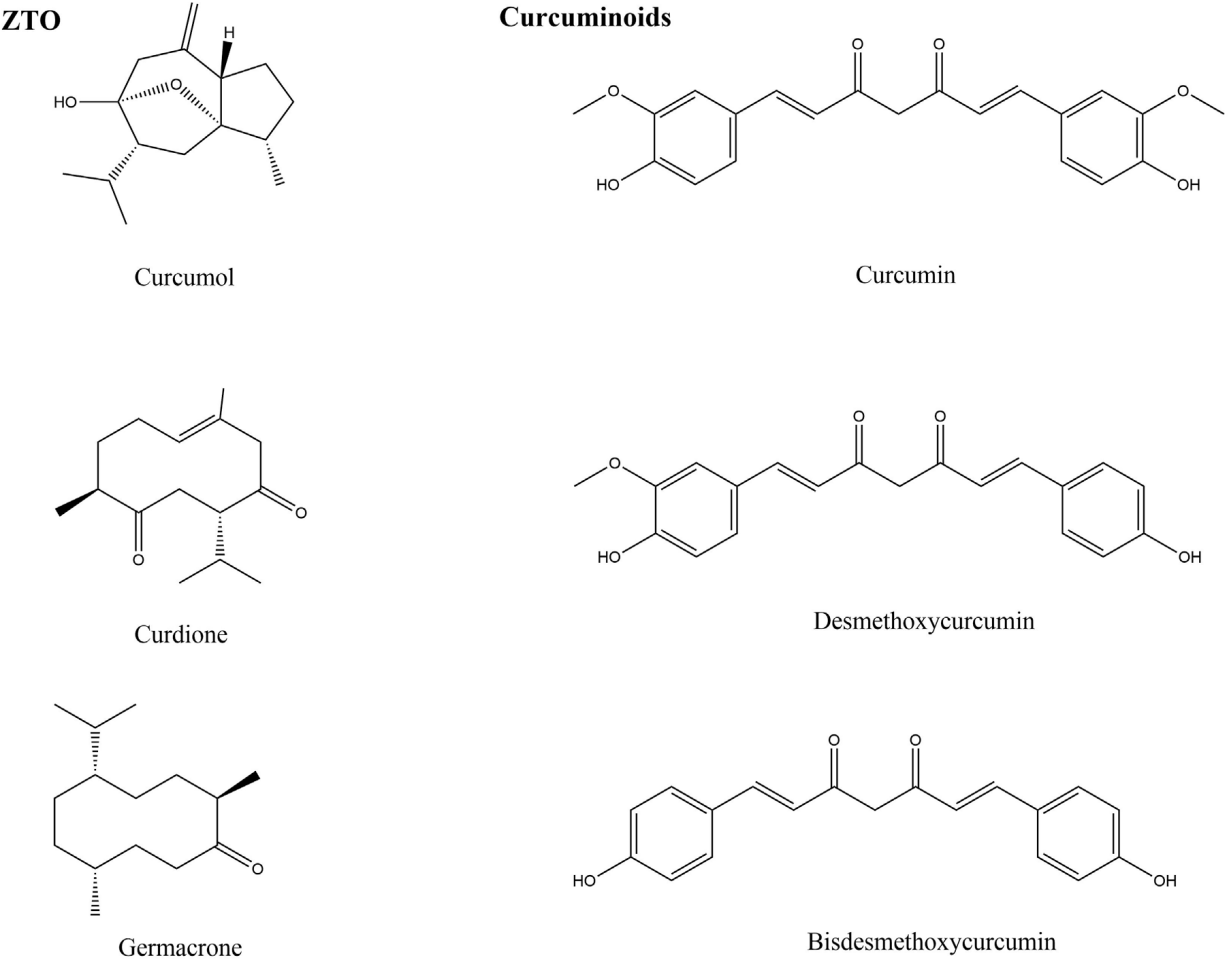
The chemical structures of active components in ZTO and curcuminoids.

Hundreds of constituents have been identified from ZTO, the main compounds with antimicrobial activity are curcumol, curdione, and germacrone. The chemical structures of these compounds are shown in Figure 1. These constituents have different curative effects and mechanisms for treating infectious diseases. Modern pharmacological studies have found that curcumol, curdione and germacrone suppress viral replication. Particularly, germacrone showed increased *in vitro* and *in vivo* antiviral therapeutic potential because it increased expression of antiviral proteins and it significantly reduced intracellular viral load (Li et al., 2020).

### 2.2 Curcuminoids

Curcuminoids are non-toxic polyphenolic derivatives (yellow-orange powder), which mainly refer to diphenylheptane hydrocarbons, but also include some amyl hydrocarbons. There are 20 types of curcumin compounds found in Curcumae Rhizoma. The main active components of curcuminoids are curcumin (77%), demethoxycurcumin (DMC, 17%), and bisdemethoxycurcumin (BDMC, 6%) (Aggarwal and Sung, 2009). The chemical structures of these curcuminoids are shown in Figure 1.

Importantly, curcumin is a lipophilic polyphenol substance and the most extensively studied component. DMC and BDMC are natural analogues of curcumin with similar biological activity to curcumin. One study found that curcumin inhibits NF-κB much more effectively than others (curcumin > DMC > BDMC), this result may be related to the important role of the methoxy group on the phenyl ring of curcumin (Sandur et al., 2007). Curcumin has been determined to be safe and tolerable by the United States Food and Drug Administration, even at a dose of 12 g per day (Devassy et al., 2015; Hewlings and Kalman, 2017).

## 3 Pharmacokinetics

Despite a long history of clinical application, Curcumae Rhizoma and its active constituents have faced many barriers due to low bioavailability after administration. Low water solubility, hydrophobicity, and poor stability lead to the poor absorption, limited tissue distribution, and extensive metabolism. Therefore, researchers are constantly looking for ways to increase the bioavailability of these constituents. Currently, the medicinal products on the market are limited to ZTO injection, freeze-dried powder, and vaginal suppositories. Therefore, more efforts are required to develop novel ZTO drug delivery systems to enhance their overall quality. To address the issue of poor water solubility and low bioavailability of ZTO and its main components *in vivo*, several studies have been conducted on nanoparticle delivery systems, which has resulted in remarkable data in the last decade (Chen et al., 2011). Liposomes (Chen et al., 2021), solid lipid nanoparticles (Yang et al., 2020), and microspheres (Song and Sun, 2016) are examples of delivery systems which have been studied. In addition, one study showed that preparation of nanocapsules of curdione using the melting method increases curdione solubility. Further, *in vitro* release experiments showed that curdione-nanocapsules have a sustained release effect (Wu et al., 2011). A pharmacokinetic study on ZTO and a ZTO-b-cyclodextrin inclusion complex in pigs indicated that Cmax, Tmax, AUC, and MRT were significantly different in the ZTO-b-cyclodextrin group compared to the ZTO group. Moreover, the relative bioavailability of ZTO-b-cyclodextrin was significantly higher compared to the ZTO group (Yong-Xue et al., 2012). An *in vitro* study of the absorption mechanism of ZTO and some monomers (i.e., curcumol, germacrone, curdione) showed that curdione had a high apparent permeability coefficient, which was greater than curcumol and germacrone (Leng et al., 2007). The structure of curcudione can be modified by micro-biological transformation technology to improve its water solubility and pharmacological activity. Thus, Ma et al., 2007 used platycodonum platyflorum suspension cells to convert curcudione and isolated 7 gemmarane sesquiterpene alcohol compounds.

Over the past 3 decades, many studies have concentrated on the absorption, metabolism, and tissue distribution of curcumin, all of which reached the conclusion that curcuminoids are poorly absorbed and metabolized quickly *in vivo*. Phase I clinical trials demonstrated poor bioavailability of curcumin even at high doses (Anand et al., 2007). The key factor affecting the effectiveness of oral doses was absorption degree and absorption speed in the digestive tract. When taken orally, most of the curcumin was passed out of the body in the stool, and only a small amount was absorbed in the intestine and rapidly metabolized in the plasma and liver. One study investigated the absorption behavior of curcumin in mice by gavage and intraperitoneal (i.p.) injection (Schiborr et al., 2010), the results showed that the curcumin concentration in the plasma, liver, and brain of mice in the gavage group (50 mg kg^−1^) were all below the detection limit, while the curcumin content in the brain of the injection group (100 mg kg^−1^) was 4–5 μg g^−1^. Compared to oral administration, i.p. injection can significantly increase plasma curcumin concentration (Pan and Wang, 2012). This suggests that the pharmacokinetic parameters may be affected by different routes of administration. Co-administrating with other drugs could also increase the bioavailability of curcumin. In one study, volunteers took 2 g curcumin on fasting for 1 h, and the plasma level of curcumin was less than 10 ng mL^−1^. However, curcumin bioavailability increased by 2,000% after co-administrating with 20 mg piperine (Amit and Ben-Neriah, 2003).

Due to its extremely low water solubility, to improve the bioavailability, prolong bioactivity, and pharmacokinetic properties *in vivo* and *in vitro*, many researchers have studied the effect of different forms of curcumin, including, but not limited to, solid dispersions, liposomes, cyclodextrin inclusion complexes, microemulsions, micelles, nanoparticles, alcohol bodies, microspheres, microcapsules, and precursor drugs (Liu et al., 2016; Ma et al., 2019; Li et al., 2021). In one study using a curcumin nanoparticle suspension, the AUC_0-∞_ of the preparation was 5.85 times higher than that of free drug and the bioavailability was significantly increased. This suggests that the bioavailability of curcumin is significantly increased by improving its water solubility (Ipar et al., 2019). Chen Jing found that DMC and BDMC, the main components of curcuminoids, had improved bioavailability compared to curcuminoids. Further, compared to DMC, the bioavailability of the DMC-nanoemulsion was significantly improved (Chen et al., 2016). Although significant progress has been achieved over the past few decades, there are still various drawbacks in improving bioavailability. Moving forward, further studies are required to investigate additional strategies that could be applied to enhance the bioavailability of Curcumae Rhizoma and its active constituents.

## 4 Pharmacological actions of Curcumae Rhizoma

### 4.1 Antibacterial effect

**FIGURE 2:**
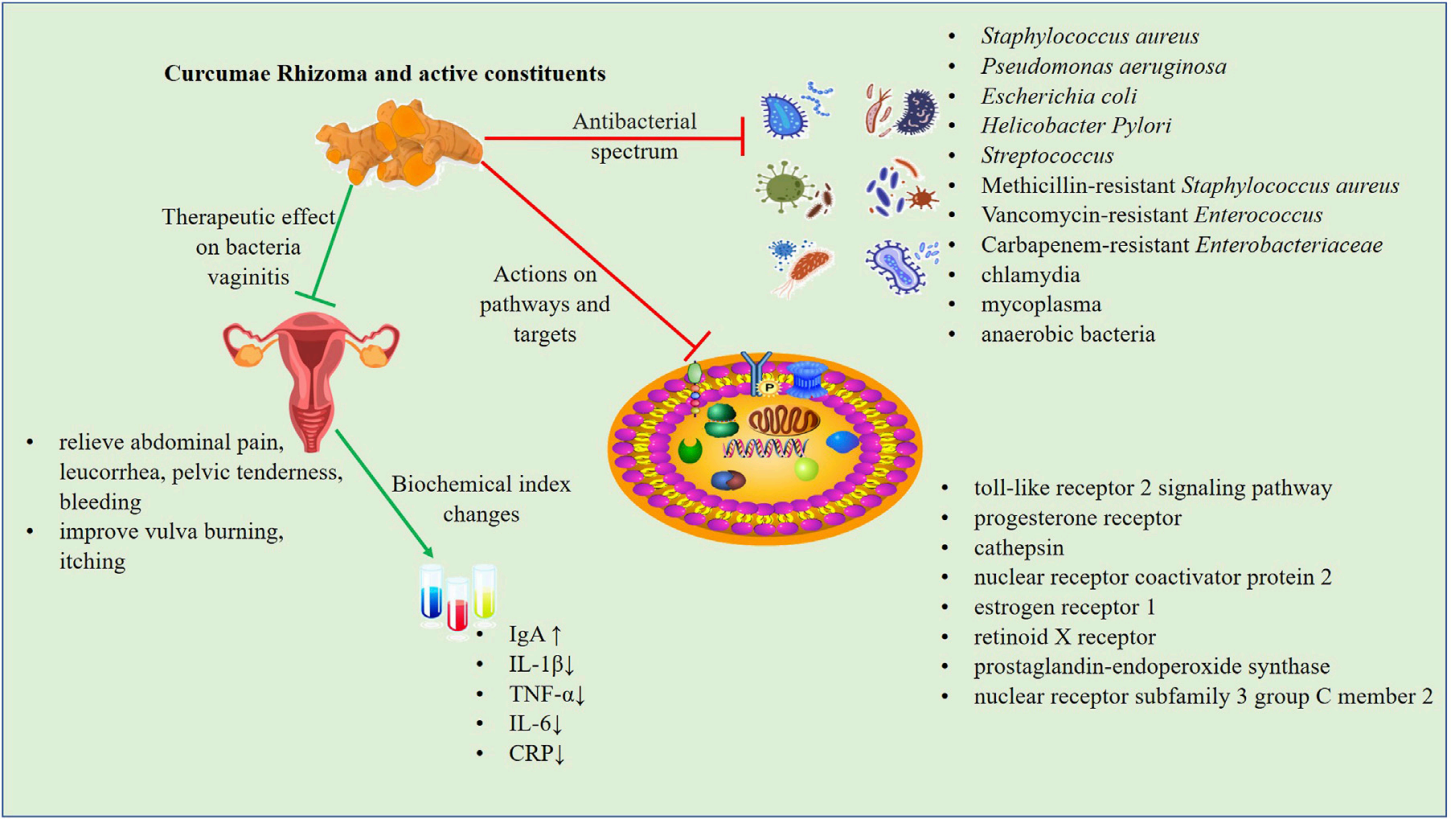
The antibacterial mechanism of Curcumae Rhizoma and its active constituents. Both Curcumae Rhizoma and its active constituents have strong antibacterial activities against a variety of bacteria through different signaling pathways and targets. The ZTO vaginal suppository has definite clinical efficacy in the treatment of bacterial vaginitis, which can significantly decrease inflammatory factors (TNF-α, IL-6, and CRP), and ameliorate clinical symptoms.

Both Curcumae Rhizoma and its active components have strong antibacterial activities against a variety of bacteria through different signaling pathways and targets. A graphical presentation of the antibacterial effect of Curcumae Rhizoma and its active constituents is provided in Figure 2.

#### 4.1.1 Curcumae Rhizoma has good antibacterial activity

Curcumae Rhizoma may be an alternative antimicrobial agent to fight fatal bacterial infections (Sindhu et al., 2011). Moreover, dried Curcumae Rhizoma powder has been used in traditional Chinese medicine to treat infections. Curcumin, a bioactive compound derived from Curcumae Rhizoma, is known for its antibacterial properties. Curcumin possesses strong antimicrobial activity against many Gram-positive and Gram-negative bacteria (Moghadamtousi et al., 2014; Teow et al., 2016; Zheng et al., 2020).

A recent study showed that curcumin had antimicrobial activity against strains of *Staphylococcus aureus* and *Pseudomonas aeruginosa*, and improved the effect of ciprofloxacin (Kamurai et al., 2020). Huang reported that curcumin presented good activity against *Escherichia coli* and *S. aureus* with MIC values of 44.4 mg L^−1^ (Huang et al., 2015). In an *in vivo* study of pylori-infected C57BL/6 mice, curcumin showed significant therapeutic potential and significantly eradicated *Helicobacter Pylori* (HP) and ameliorated gastric damage caused by HP. In addition, curcumin also played a significant role against 65 clinical *HP* strains in the absence of an antimicrobial, with the minimum MIC values ranging from 5 to 50 μg/mL (De et al., 2009). Interestingly, Curcumae Rhizoma mouthwash can be successfully used as an adjunct to mechanical plaque management measures to prevent plaque and gingivitis, and it significantly reduces total microbial load (Waghmare et al., 2011).

The resistance of pathogenic bacteria to various antibiotics has led to the need for new and effective strategies to tackle antimicrobial resistance (El-Kattan et al., 2022). In clinical settings, common multidrug-resistant bacteria include Methicillin-resistant *S. aureus* (MRSA), Vancomycin-resistant *Enterococcus* (VRE), ultra-broad spectrum *β*-lactamase (ESBLs), and Carbapenem-resistant *Enterobacteriaceae*, among others, which pose a significant threat to patients (Wang Z. et al., 2022). An *in vitro* study testing the antibacterial and synergistic effects of curcumin against MRSA strains showed that curcumin had strong antibacterial activity and synergistic effects either alone or combination with certain antibiotics, namely oxacillin, ampicillin, ciprofloxacin, and norfloxacin) (Mun et al., 2013). Additionally, another study demonstrated the synergistic effect of curcumin with antibiotics (e.g., imipenem and colistin) on Carbapenem-resistant *Enterobacteriaceae in vitro* and activity against *Galleria mellonella* larvae *in vivo* (Ozturk, 2022). Those studies reveal the potential role of curcumin on multi-drug resistance and the possibility to drastically reduce the use of existing antibiotics.

#### 4.1.2 ZTO vaginal suppository is effective at treating bacteria vaginitis

The ZTO vaginal suppository contains ZTO and borneol. The preparation can promote vaginal epithelial cell proliferation by inhibiting the proliferation of hemolytic *Streptococcus* and *S. aureus* in the vagina and can also enhance the self-cleaning function of the vagina, improve vaginal resistance to infection, and vaginal microcirculation (Peng, 2019). As a byproduct of Curcumae Rhizoma, ZTO is effective against *S. aureus*, *Streptococcus, E. coli, P. aeruginosa*, *chlamydia*, *mycoplasma*, and anaerobes (Negi et al., 1999; Peng, 2019; Zeng, 2019).

In the treatment of bacterial vaginitis, Wang (2022) found that ZTO vaginal suppository combined with azithromycin (1 g/day) had a better effect than oral azithromycin dispersive tablets alone. This combination treatment ameliorated pH, bacterial community concentration, bacterial community diversity, increased the level of IgA, and decreased interleukin (IL)-1β expression. Another study on the treatment of bacterial vaginitis combined with pelvic inflammation by ZTO vaginal suppository showed that the relief time of lower abdominal pain, abnormal leucorrhea, pelvic tenderness, and vaginal bleeding were shortened. Further, TNF-α, IL-6, and CRP were decreased, the Pittsburgh Sleep index (PSQI) score was lower than pre-treatment, and the Short Form Health Life Scale (SF-36) score was higher than before treatment (Lan and Yang, 2022). Therefore, the ZTO vaginal suppository has definite clinical efficacy in the treatment of bacterial vaginitis with pelvic inflammatory disease, which can effectively decrease inflammatory factors, shorten the time of symptom improvement, and improve the quality of life of affected individuals. As women age ovarian function declines, leading to a reduction in the secretion of female hormones, resulting in vaginal wall atrophy and damage to the resistance to vaginal function. This may lead to a situation whereby infection with pathogenic bacteria, including *E. coli* and *Staphylococcus,* happens more readily. Co-administration of a ZTO vaginal suppository with estrogen ointment has been shown to have a synergistic effect and also improved vulva burning, itching, and other symptoms (Zhang et al., 2017). Mixed infectious vaginitis refers to two or more types of mixed infection, partly for *chlamydia*, *mycoplasma*, anaerobic bacteria, and aerobic bacteria mixed infection (Zeng, 2019). In addition, studies have shown that the clinical efficacy of *lactobacillus* vagina capsule combined with roxithromycin and ZTO vaginal suppository was relatively accurate, and the recurrence rate was effectively reduced in patients with mixed infectious vaginitis.

In mechanistic studies, ZTO was found to attenuate or prevent the strong inflammatory reaction of the vaginal mucosa by downregulating the toll-like receptor 2 (TLR-2) signaling pathway, leading to a reduction in inflammation and improved function of the vaginal mucosa (Li et al., 2021). Moreover, the potential regulatory network of Curcumae Rhizoma against vaginitis was preliminarily revealed through network pharmacology. Curcumae Rhizoma has been shown to directly act on progesterone receptor (PGR), cathepsin (CTSD), nuclear receptor coactivator protein 2 (NCOA2), estrogen receptor (ESR1), retinoid X receptor (RXRA), and other protein targets, and achieve its therapeutic purpose by regulating estrogen and thyroid hormone signaling pathways (Huang et al., 2019). In another network pharmacological study, it was revealed that the key targets of Curcumae Rhizoma include PGR and ESR1, as well as prostaglandin-endoperoxide synthase 1 (PTGS1), prostaglandin-endoperoxide synthase 2 (PTGS2), and nuclear receptor subfamily three group C member 2 (NR3C2) (Zhen et al., 2020). This suggests that the pharmacological anti-bacterial vaginitis effects of Curcumae Rhizoma may be achieved through multi-component, multi-target, and multi-pathway mechanisms.

### 4.2 Antiviral effect

Curcumae Rhizoma and its active components have remarkable antiviral properties, and the associated mechanism(s) of action are complex and have been investigated in depth. We collected data from different studies, including those conducted *in vitro*, *in vivo*, in clinical trials, as well as network pharmacology studies. These studies all had a focus on the activity, performance, and mechanism of Curcumae Rhizoma and its active components in viruses (herpesvirus, hepatitis B virus, human immunodeficiency virus, and human papilloma virus) and virosis (COVID-19 and respiratory infection diseases). The specific mechanisms of action are shown in Table 2.

**TABLE 2:** Antiviral activities of Curcumae Rhizoma and its active constituents.

Virus	Antiviral substances	Description of antiviral activity type	References
COVID-19	Turmeric	Improve the severity of clinical symptoms (e.g., cough, anosmia and ageusia)	Chabot and Huntwork, (2021)
COVID-19	ZTO injection	Promote absorption of lung lesions, reduce lung injury, and reduce the fatality rate in severe patients	Jiang et al. (2020)
COVID-19	Curcumae Rhizoma	The mechanism of action of Curcumae Rhizoma against COVID-19 may be related to inhibition of inflammatory molecules	Qin et al. (2020)
COVID-19	Curcuminoids	Curcuminoids is an excellent candidate for COVID-19 prevention and treatment	Fu et al. (2022)
COVID-19	Curcumin	Curcumin blocks SARS-CoV-2 entry into cells as well as viral replication; both bromelacin and curcumin have anti-inflammatory properties including inhibition of transcription factors, leading to subsequent downregulation od pro-inflammatory mediators	Kritis et al. (2020)
COVID-19	Curcumin	Curcumin exhibits good binding affinity at the active site of the SARS-CoV-2 RdRP complex	Singh et al. (2021)
COVID-19	Turmeric	Turmeric has a potential to inhibit the SARS-CoV-2 vital proteins	Emirik, (2022)
COVID-19	ZTO	ZTO shows strong dose-dependent inhibitory effects on SARS-CoV-2 virus	Zhou et al. (2022)
COVID-19	Curcumin	Curcumin inhibits trans-activation of various AIDS-related kinases, including tyrosine kinase, protein kinase 1, mitogen-activated protein kinase, protein kinase C, and cyclin kinase	Prasad and Tyagi, (2015)
COVID-19 (pulmonary fibrosis)	Curcumin and Curcumenol	Effectively regulate the expression of TGF-β1, α -smooth muscle actin (α-SMA) and collagen-III; reduces extracellular matrix and collagen fiber deposition, and relieves bleomycin-induced pulmonary fibrosis in mice; promoting the expression of autophagy related proteins LC3B-II and Beclin1 and inhibiting the PI3K/Akt/mTOR signaling pathway may	Hu et al. (2020)
COVID-19 (pulmonary fibrosis)	Astragalus-Turmeric Mixture	Astragalus-Turmeric Mixture can significantly inhibit bleomycin-induced pulmonary fibrosis in rats, and inhibition of TGF-β1 mRNA expression is one of the possible mechanisms	Sun et al. (2007)
COVID-19	Curcumin	Regulate the intracellular signal transduction pathways involved in inflammation, including IBB, NF-kBERK1,2, AP-1, TGF-β, TXNIP, STAT3, PPARγ, JAK2-STAT3, NLRP3, p38MAPK, Nrf2, Notch-1, AMPK, TLR-4 and MyD-88	Saeedi-Boroujeni et al. (2021)
Influenza virus	ZTO	The effects of ZTO against influenza virus have been confirmed	Xia et al. (2004)
Influenza virus	ZTO	ZTO has a direct inactivation effect to influenza virus A1 and influenza virus A3, which can restore the patient’s body temperature to normal and relieve symptoms such as cough and sore throat	Lin, (2011)
Influenza virus (H1N1)	ZTO	ZTO inhibits the replication of H1N1	Li et al. (2020)
Influenza virus (H3N2)	ZTO	ZTO inhibits the replication of H3N2	Liao et al. (2013)
Influenza virus (H5N1)	ZTO	ZTO inhibits the replication of H5N1	Cai et al., 2020
			Huang et al. (2009)
Influenza virus	ZTO combined with oseltamivir	Has significantly effect in treatment of children with viral pneumonia, significantly decreased the serum levels of IL-8, C-reactive protein, creatine kinase, and cardiac troponin	Wang et al. (2019b)
Influenza virus (H5N1)	The active components of ZTO (curcumol and hypericin)	Inactivate H5N1 virus on MDCK cells, block virus infection, inhibit virus adsorption, and treat virus invaded cells	Huang et al. (2009)
Influenza virus	Curcumin	Curcumin inhibits influenza viruses by disrupting the integrity of the viral envelope and liposome membranes	Chen et al. (2013)
Influenza virus (H1N1, H5N1)	ZTO	Curcumol, curdione, and germacrone, which are the three main active ingredients from ZTO, inhibit the replication of H1N1 effectively, among which germacrone has the best effect	Li et al. (2020)
Influenza virus (H1N1, H3N2, influenza A and influenza B viruses)	The active components of ZTO (germacrone)	germacrone inhibited the attachment/entry step and early stage of replication cycle of H1N1, H3N2, influenza A and influenza B viruses by inhibiting the expression of viral proteins and RNA synthesis of progeny viruses on MDCK and A549 cells	Liao et al. (2013)
Influenza virus (H1N1 and H6N1)	Curcumin	Directly affects replication of H1N1 and H6N1 subtypes of influenza virus by inhibiting hemagglutinin	Chen et al. (2013)
Influenza virus	Curcumin	Curcumin may interfere with influenza virus entry by interacting with hemagglutination protein in the receptor-binding region of the virus	Ou et al. (2013)
Influenza virus	Anti-influenza active ingredients of Curcumae Rhizoma	May be achieved by regulating phosphatidylinositol-3-hydroxykinase (PI3K) protein kinase B (Akt) and tyrosine kinase (Jak)-transcription factor (STAT) signaling pathway. The effective components of Curcuma longa L have binding and inhibitory effects on influenza virus protein targets and inflammatory related targets, which indicate the pattern of multi-targeting anti-influenza action	Ti et al. (2021)
RSV	Antiviral oral liquid	Has a remarkable effect on respiratory diseases caused by RSV infection	Liu, (2018)
RSV	ZTO	The pulmonary index value also decreased correspondingly, presenting a significant dose correlation	Xia et al. (2004)
RSV	Uniform and stable silver nanoparticles (AgNPs) with antiviral properties by using curcumin	Had an efficient inhibitory effect on respiratory syncytial virus infection and had no toxicity to host cells; AgNPs can directly inactivate RSV and prevent RSV from infecting host cells	Yang et al. (2016)
Adenovirus	Curcumin	Reduced adenovirus replication, gene expression, and virus production at curcumin concentrations that had little effect on cell viability	Jennings and Parks, (2020)
HSV	Curcumin	Curcumin affects vp16-mediated recruitment of RNA polymerase II to the IE gene promoter through a mechanism that depends on the activity of histone acetyltransferases of p300/CBP, leading to suppression of gene expression and blocking viral infection	Kutluay et al. (2008)
HSV-2	Curcumin	Curcumin effectively inhibited HSV-2 replication	Bourne et al. (1999)
HSV-2	Curcumin	Inhibiting HSV-2 replication is related to the transcription factor NF-κB	Flores et al. (2016)
HSV-2	Curcumin nanoparticles	Inhibit HSV-2 infection and replication	Vitali et al. (2020)
HBV	Aqueous extract of Curcumae Rhizoma	Increased the level of p53 protein *in vitro*, which inhibits the HBV enhancer and X promoter that mediate HBV replication	Kim et al. (2009)
HBV	Curcumae Rhizoma	Reduce the expression of HBx in the liver *in vivo*, increase the expression of p21, cyclin D1 and the level of p-p53; effect on the early and late stages of liver disease, and prevent and delay the occurrence of liver cancer	Kim et al. (2011)
HBV	Curcumin	Downregulating the acetylation of CCcdNA-bound histones, thereby inhibiting HBV gene replication	Wei et al. (2017)
HBV	Curcumin exerts	Targeting multiple cellular and molecular pathways such as Wnt/β-catenin, Ap1, STAT3, MAPK and NF-κB signaling pathways	Hesari et al. (2018)
HBV	KCT-01 (a new botanical drug formula containing Curcumae Rhizoma)	Inhibit HBV replication and IL-6 *in vitro* and *in vivo* models without showing toxicity, indicating that KCT-01 potential as an antiviral drug alone or in combination with entecavir	Kim et al. (2018)
HIV	Curcumin and curcumin A	Curcumin and curcumin A exhibit similar inhibitory effects on of HIV-1 infection in cultured lymphoblastoid T cells; curcumin A inhibited HIV-1 reverse transcription at low concentrations; curcumin A induced HO-1 expression and reduced the progression of the HIV-1 infection cycle	Kumari et al. (2015)
HIV/AIDS	Oral curcumin	Oral curcumin supplementation positively regulates energy metabolism in HIV/AIDS patients receiving antiretroviral therapy	da Silva et al. (2019)
HIV	Curcumin	No antiviral effect in clinical trial, but patients prefer to take curcumin to endure mild gastrointestinal pain and feel better	Pawar et al. (2021)
HIV	Curcumin	Inhibition of LTR activity to block HIV-1 replication and inhibits HIV-1 LTR-directed gene expression	Nabel et al. (1988)
			Li et al. (1993)
HIV	Curcumin	Inhibit trans-activator of transcription protein transactivation of the HIV1-LTR genome, inflammatory molecules (interleukins, TNF-α, NF-κB, COX-2) and HIV associated various kinases including tyrosine kinase, PAK1, MAPK, and PKC.	Prasad and Tyagi, (2015)
HIV	Turmeric	Inhibit nucleocapsid proteins which play a key role in controlling HIV viral replication	Wang et al. (2021)
HPV	ZTO vaginal suppository	The total effective rate and the recovery rate were improved significantly	Wang et al. (2022b)
			Hu et al. (2021)
HPV	ZTO vaginal suppository	Significantly decreased HPV-DNA viral load, down-regulated IL-6, TNF-α, hs-CRP, and increased CD3^+^, CD4^+^, CD8^+^ and CD4+/CD8+ water level	Hu et al. (2021)
HPV	ZTO vaginal suppository	Execute its anti-HPV activity primarily by decreasing the expression of HPV16E6E7 in cervical cancer cell line and inhibit the growth of cervical epithelial cell	Zhang et al. (2007)
HPV	ZTO vaginal suppository	Regulation of immunity and the reduction of inflammatory response	Yin et al. (2021)
HPV	Curcumin	Selectively downregulate HPV18 transcription and AP-1 binding activity in HeLa cells; the complete down-regulation of AP-1 binding activity and reversal of c-fos/FRA1 transcription to normal state was observed in tumorigenic HeLa cells mediated	Prusty and Das, (2005)
HPV	Curcumin	Selectively inhibit the expression of viral oncogenes E6 and E7; downregulated activation of NF-kB induced by TNF-a; blocks the phosphorylation and degradation of IkBa; down-regulate the expression of NFkB-regulated COX-2 gene, and selectively down-regulate AP-1 binding	Divya and Pillai, (2006)

#### 4.2.1 Respiratory infection

##### 4.2.1.1 Influenza virus

Influenza virus is one of the most commonly detected viruses around the world. Human influenza viruses are classified into types A, B, and C, with influenza A virus causing the most damage. Influenza A viruses are further divided into subtypes based on different proteins expressed on the surface of the viruses, including H1N1, H3N2, H5N1, and H7N9 (Zhou et al., 2015).

In China, many Chinese patent medicines, which claim to treat influenza viruses, contain Curcumae Rhizoma. Further, oral solutions extracted from Curcumae Rhizoma have been shown to resist influenza A virus and parainfluenza II virus (Mao et al., 2018). *In vivo* and *in vitro* studies have confirmed the effect of Curcumae Rhizoma oil against influenza virus (Xia et al., 2004). Clinical studies have also shown that ZTO has a direct inactivation effect on influenza virus A1 and influenza virus A3, which restores the patient’s body temperature to normal and relieves symptoms such as cough and sore throat (Zhang et al., 2007; Lin, 2011). In some reports, ZTO has been shown to inhibit replication of H1N1 (Li et al., 2020), H3N2 (Liao et al., 2013), and H5N1 (Huang et al., 2009; Cai et al., 2020). Further, ZTO has also been shown to have an effect in children with viral pneumonia by significantly decreasing serum levels of IL-8, CRP, creatine kinase, and cardiac troponin (Wang L. et al., 2019).

*In vitro* studies have demonstrated that curcumin extract directly kills H1N1 and H3N2 subtypes in Madin-Darby canine kidney (MDCK) cells (Lai et al., 2020; Dound and Sehgal 2021). In mice with influenza virus model, curcumin (25–100 mg/kg) has been shown to improve the degree of lung lesions, reduce the lung index, significantly prolong the average survival days of infected mice, and decrease mortality (Xu et al., 2009). In addition, the active component of ZTO was found to block viral infection, inhibit viral adsorption, and prevent viral invasion into cells, thus inactivating the H5N1 virus in MDCK cells. (Huang et al., 2009). Furthermore, Chen showed that curcumin inhibits influenza viruses by disrupting the integrity of the viral envelope and liposome membranes (Chen et al., 2013). Furthermore, Li and others confirmed that curcumol, curdione, and germacrone, which are the three main active ingredients from ZTO, can effectively inhibit H1N1 replication, among which germacrone has the best effect (Li et al., 2020). Liao et al. (2013) reported that germacrone inhibits the attachment/entry step at the early stage of the replication cycle of H1N1, H3N2, influenza A, and influenza B viruses by inhibiting the expression of viral proteins and RNA synthesis of progeny viruses in MDCK and A549 cells (Liao et al., 2013). In addition, there is growing evidence to suggest that curcumin directly affects the replication of influenza viruses by inhibiting hemagglutinin, or by interfering with the entry of influenza virus by interacting with hemagglutinin in the receptor binding region of the virus (Chen et al., 2013; Ou et al., 2013).

Based on systems pharmacology studies, Ti et al. (2021) discovered that the anti-influenza activities of Curcumae Rhizoma may be achieved by regulating phosphatidylinositol-3-hydroxykinase (PI3K) protein kinase B (Akt) and tyrosine kinase (Jak)-transcription factor (STAT) signaling pathways. The effective components of Curcumae Rhizoma have a binding and inhibitory effect on influenza virus proteins and inflammatory related targets, which indicate the pattern of multi-targeting anti-influenza actions (Ti et al., 2021).

##### 4.2.1.2 Respiratory syncytial virus (RSV) and adenovirus

Respiratory syncytial virus (RSV) is a common pathogen that causes viral pneumonia in children. Antiviral oral liquid, which is composed of Curcumae Rhizoma, anemone, Rehmannia glutinosa, and patchouli, combined with chemical drugs, have a prominent effect on respiratory diseases driven by RSV infection (Liu, 2018). In mice experiments have demonstrated that ZTO injection has an inhibitory effect on RSV. Moreover, an increase in the dose (20, 40, 80 mg/kg) leads to an enhanced therapeutic effect as well as a reduction in the pulmonary index value, presenting a significant dose correlation (Xia et al., 2004). Some researchers have prepared uniform and stable silver nanoparticles (AgNPs) with antiviral properties using curcumin, which has an efficient inhibitory effect on RSV infection and had no toxicity to host cells. Mechanistic studies showed that AgNPs could directly inactivate RSV and prevent viral infection of host cells, which make this a potentially promising novel highly effective RSV fungicide (Yang et al., 2016).

Adenoviruses are double-stranded DNA non-enveloped viruses, which can infect the respiratory tract, intestines, eyes, and liver. Adenoviruses can also cause respiratory infection in children and with high mortality. A study focusing on the effect of curcumin on adenovirus replication demonstrated that treatment of cultured cells with curcumin reduced adenovirus replication, gene expression, and virus production, while having little effect on cell viability. Thus, curcumin represents a promising compound for further investigation as a potential treatment for adenovirus infections (Jennings and Parks, 2020).

##### 4.2.1.3 COVID-19

Since COVID-19 emerged as a global public health crisis, many agents have been studied as potential treatments for the disease. As a novel coronavirus, SARS-CoV-2 showed a high degree of sequence similarity with SARS (Wang et al., 2020; Emirik, 2022). During the SARS pandemic, Curcumae Rhizoma combined with Western medicine was used in patients with the asthmatic stage of the disease and showed good therapeutic effect. An *in vitro* study showed that ZTO had a strong dose-dependent inhibitory effects on the SARS-CoV-2 virus (Zhou et al., 2022). In patients with COVID-19, Curcumae Rhizoma and its active constituents (ZTO and curcumin) significantly improved the severity of clinical symptoms, such as cough, anosmia, and ageusia (Chabot and Huntwork, 2021) to promote absorption of lung lesions and reduce lung injury leading to a significant reduction in mortality in severe patients (Jiang et al., 2020).

Qin et al. (2020) hypothesized that the mechanism of action of Curcumae Rhizoma against COVID-19 may be related to inhibition of inflammatory molecules. Multiple lines of evidence suggest that curcumin, which is isolated from Curcumae Rhizoma is an excellent candidate for COVID-19 prevention and treatment (Fu et al., 2022). One study highlighted the potential therapeutic role of the combination of bromelacin and curcumin in preventing COVID-19. This study showed that curcumin blocks SARS-CoV-2 entry into cells as well as viral replication, inhibits transcription factors, and downregulates pro-inflammatory mediators (Kritis et al., 2020). RNA dependent RNA polymerase (RdRP) is a defined molecular target for inhibition of SARS-CoV-2. Interestingly, Prasad and Tyagi found that curcumin inhibits trans-activation of various Acquired Immune Deficiency Syndrome (AIDS)-related kinases, including tyrosine kinase, protein kinase 1, mitogen-activated protein kinase, protein kinase C, and cyclin kinase (Prasad and Tyagi, 2015). This explains why anti-AIDS drugs, such as lopinavir and ritonavir, were found to be effective against COVID-19 during the early stages of the pandemic (Saeedi-Boroujeni et al., 2021).

The mechanism of COVID-19 treatment may also be related to the anti-pulmonary fibrosis effect. Curcumae Rhizoma may play a prominent role in improving cough, fever, lung rales, and the degree of lung function injury by anti-pulmonary fibrosis (Qiao et al., 2020). *In vivo* studies with bleomycin-induced pulmonary fibrosis mice, curcumin was shown to be highly effective at regulating the expression of TGF-β1, *α*-smooth muscle actin (α-SMA), and collagen-III, as well as in the reduction of extracellular matrix and collagen fiber deposition (Sun et al., 2007; Hu et al., 2020). *In vitro* studies also showed that curcumin promotes the expression of autophagy related proteins LC3B-II and Beclin1, and inhibits the phosphatidylinositol-3-kinase/Akt/mammalian target of the rapamycin (PI3K/Akt/mTOR) signaling pathway, which is one of the most important signal transduction mechanisms in alleviating pulmonary fibrosis (Di Tu et al., 2020; Yu et al., 2020).

**FIGURE 3:**
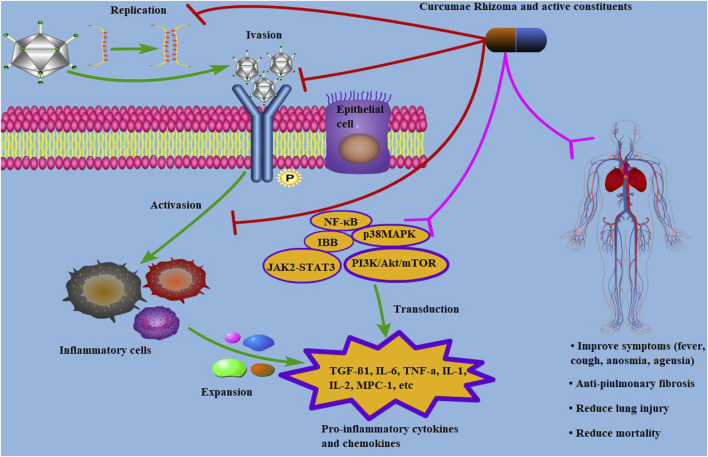
Mechanism of Curcumae Rhizoma and its active constituents against COVID-19. Antiviral mechanism of Curcumae Rhizoma and its active constituents in treating with COVID-19. Curcumae Rhizoma and its active constituents exhibit effects on COVID-19 by “multi-component, multi-target, multi-pathway”. The steps include preventing viral replication and invasion, suppressing the activations of inflammatory cells, and regulating the signaling pathways by acting on the targets, leading to improvement of respiratory symptoms.

As a key player in signal transduction pathways within inflammatory cells, curcumin regulates multiple targets involved in intracellular signal transduction pathways of inflammation, signal transducers and activators including PI3K, ERK1, AP-1, TGF-β, TXNIP, STAT3, PPARγ, JAK2-STAT3, p38MAPK, and AMPK, as well as myeloid differentiation primary response gene 88 (MyD-88). Curcumin also downregulates the expression of inflammatory enzymes such as COX2 and inducible nitric oxide synthase (iNOS), as well as CRP and inflammatory factors such as TNF-α, IL-6, and IL-8 (Patel et al., 2020; Saeedi-Boroujeni et al., 2021). Therefore, one hypothesis suggests that Curcumae Rhizoma and its active constituents act on COVID-19 through multi-targets and multi-inflammatory signaling pathways (Figure 3).

#### 4.2.2 Herpesvirus (HSV)

Herpesvirus (HSV) is a large DNA virus that commonly infects humans and animals, which has the capacity to induce latent and lytic infection in the host, leading to serious health complications. There are two types of HSV: HSV-1 and HSV-2. HSV-1 can be widely spread by mouth-to-mouth contact, causing herpes labialis, herpetic whitlow, keratitis, and genital herpes. Genital herpes is a common sexually transmitted disease caused by HSV-2. Considering the antiviral capabilities of curcumin, the main biological functions of which is to defeat viral infections by targeting viral entry and ingredients critical for viral replication and other cellular or molecular processes involved in the viral life/infection cycle.

The mechanism of curcumin attributable to the action of anti-HSV effects has been thoroughly investigated through various *in vivo* and *in vitro* experiments (LoTempio et al., 2005; Kutluay et al., 2008). These mechanisms are associated with curcumin’s capacity to block a series of cellular and molecular events which are essential for viral replication, viral gene expression, and pathogenesis. Curcumin affects vp16-mediated recruitment of RNA polymerase II to the IE gene promoter through a mechanism that depends on the activity of histone acetyltransferases of p300/CBP, leading to suppression of gene expression and viral infection (Kutluay et al., 2008). Preclinical studies have shown that curcumin effectively inhibits HSV-2 replication (Bourne et al., 1999). The mechanism of curcumin has been revealed by inhibition of HSV-1 and HSV-2 adsorption *in vitro* (Flores et al., 2016). Other mechanisms have also confirmed that curcumin inhibits HSV-2 replication is related to the transcription factor, NF-κB (Ferreira et al., 2015). Inhibition of HSV-2 infection and replication by curcumin nanoparticles has been suggested as one of the mechanisms responsible for their anti-inflammatory properties (Vitali et al., 2020). Thus, more studies are required to fully elucidate these mechanisms.

#### 4.2.3 Hepatitis B virus (HBV)

Hepatitis B virus (HBV) affects millions of people worldwide, sometimes dramatically evolving into chronic invasive infections, cirrhosis, and hepatocellular carcinoma. In China, Curcumae Rhizoma has been used as a traditional medicine to treat liver disease caused by HBV infection (Ailioaie and Litscher, 2020).

Kim et al. (2009) found that Curcumae Rhizoma extract increased the level of p53 protein *in vitro*, which inhibits the HBV enhancer and X promoter that mediate HBV replication. Furthermore, Curcumae Rhizoma was also found to reduce HBV-X protein (HBx) expression in the liver of HBx transgenic mice *in vivo*, increase the expression of p21, cyclin D1, and the level of p-p53. Taken together, these data imply that Curcumae Rhizoma has an effect on the early and late stages of liver disease, and that it prevents and delays the occurrence of liver cancer. Therefore, Curcumae Rhizoma should be considered as a potential chemo-preventive agent for HBV-related hepatocarcinogenesis (Kim et al., 2011).

Previous studies have shown that curcumin has important pharmacological effects in HBV (Rechtman et al., 2010). Experimental studies highlight the potential of curcumin as an antiviral drug *via* its downregulation of the acetylation of CCcdNA-bound histones, thereby inhibiting HBV gene replication (Wei et al., 2017). More recent studies suggest that curcumin exerts therapeutic effects on HBV patients by targeting multiple cellular and molecular pathways such as Wnt/β-catenin, Ap1, STAT3, MAPK, and NF-κB signaling pathways (Hesari et al., 2018). Kim and his team revealed that KCT-01 (a new botanical drug formula containing Curcumae Rhizoma) inhibits HBV replication and inflammatory cytokine production (IL-6) *in vitro* and *in vivo* with no toxicity, indicating the potential of KCT-01 as an antiviral drug alone or in combination with entecavir (Kim et al., 2018). However, whether curcumin can inhibit HCV replication remains unknown. A recent study found that curcumin reduces HCV gene expression by inhibiting the activation of Akt-sterol regulatory element-binding proteins-1. This suggest that curcumin inhibits HCV replication *in vitro* and may act as a new anti-HCV agent (Kim et al., 2010).

#### 4.2.4 Human immunodeficiency virus (HIV)

The antiviral effectiveness of curcumin against HIV has been studied extensively. One *in vitro* study evaluated the biological effectiveness on curcumin activity by comparing it to the synthetic analogue, curcumin A. The aim of this study was to assess HIV-1 activity at specific stages of replication (Kumari et al., 2015). The results revealed that curcumin and curcumin A produce similar inhibitory effects on HIV-1 activity in cultured lymphoblastoid T cells. HIV-1 reverse transcription was restrained by curcumin A at low concentrations, but had no role in long terminal repeats (LTRs), trans-activator of transcription protein, or transcription factor NF-κB. The same experiments were performed in lymphoblastoid cultures and the results showed that curcumin A induced HO-1 expression and slowed the progression of the HIV-1 infection cycle. By analyzing this group of activities, the authors concluded that the maintenance of anti-HIV-1 properties was associated with improved stability of curcumin A compared to curcumin, enabling its selection as an antiretroviral candidate.

In one case study, a 42 years old female infected with HIV and receiving antiretroviral treatment was evaluated for the impact of curcumin supplementation on her energy metabolism (da Silva et al., 2019). Improvements in lipid profile and insulin sensitivity, as well as positive regulation of substrate oxidation at rest, were observed during intervention with curcumin. Therefore, oral curcumin supplements positively regulate energy metabolism in HIV patients with antiretroviral therapy. In a clinical trial involving in 40 patients over 8 weeks who were treated with curcumin as anti-HIV compound, patients claimed that they preferred to take curcumin to endure mild gastrointestinal pain and feel better (Pawar et al., 2021).

Viral LTRs are implicated in the transcription of the HIV-1 provirus. Inhibition of LTR activity may be a possible pathway for antiviral agent candidates to block HIV-1 replication (Nabel et al., 1988). Curcumin is a potent compound that inhibits HIV-1 LTR-directed gene expression without any significant effect on cell viability (Li et al., 1993). Recent studies show that curcumin inhibits trans-activator of transcription protein transactivation of the HIV1-LTR genome, inflammatory molecules (interleukins, TNF-α, NF-κB, COX-2) and various HIV associated kinases including tyrosine kinase, PAK1, MAPK, and PKC (Prasad and Tyagi, 2015). In addition, Curcumae Rhizoma inhibits nucleocapsid proteins which play a key role in controlling HIV viral replication (Wang et al., 2021).

#### 4.2.5 Human papilloma virus (HPV)

Cervical cancer is the third most common female cancers in the world. It is highly connected to infection with the HPV. Among the 200 HPV types, more than 50% and 13% of cervical cancers are associated with HPV 16 and 18, respectively (Lim et al., 2010). In clinical studies, it was found that the recovery rate and total effective rate of ZTO vaginal suppository group in the treatment of HPV infection were clearly higher than the blank control group (Wang Q. et al., 2022). Recently, A meta-analysis indicated that the total effective rate and the recovery rate following treatment with a ZTO vaginal suppository was better than interferon-α2b in cervical HPV infection therapy. This treatment markedly reduced HPV-DNA viral load, downregulated IL-6, TNF-α, hs-CRP, and increased CD3^+^, CD4^+^, CD8^+^, and CD4^+^/CD8^+^ expression (Hu et al., 2021). Further, *in vitro* experimental studies have demonstrated that ZTO vaginal suppositories execute their anti-HPV activity primarily by decreasing HPV16E6E7 expression in cervical cancer cells to inhibit cervical epithelial cell proliferation (Zhang et al., 2007). The mechanism behind this might be linked to the regulation of immunity and the reduction of inflammatory responses (Yin et al., 2021).

Transcription factor AP-1 plays an essential role in the transcriptional regulation of specific types of high-risk human papillomaviruses (HPV16 and HPV18) which are etiologically associated with the development of cervical cancer in women. Prusty and Das (2005) reported that AP-1 had high binding activity, and that most of its members were highly expressed in tumor tissues, but c-fos expression gradually increased while fra1 expression decreased, which was consistent with the progression of cervical lesions. Moreover*, in vivo* and *in vitro* studies have demonstrated that curcumin selectively downregulates HPV18 transcription and AP-1 binding activity in HeLa cells.

Infection with high-risk HPV causes cervical cancer, mainly through the action of viral oncoproteins E6 and E7. Importantly, curcumin has been shown to selectively inhibit the expression of these viral oncogenes by RT-PCR and western blotting. Electrophoretic mobility shift assays have shown that curcumin downregulates activation of TNF-α-induced NF-κB, and that curcumin also inhibits the phosphorylation and degradation of IkBa, resulting in ineffective NF-κB activation. In addition, curcumin downregulates the expression of the NF-κB-regulated COX-2 gene, and selectively downregulates AP-1 binding (Divya and Pillai, 2006). Singh and his teammates speculated that the possible molecular targets of curcumin in cervical cancer cells include inhibition of telomerase, inhibition of Ras, ERK pathway finally resulting in inhibition of cyclin D1, c-Myc, Hsp 70, activation of AIF, release of cytochrome c, and triggering of apoptosis *via* the mitochondrial pathway (Singh and Singh, 2009).

### 4.3 Fungal

Curcumae Rhizoma has gained increased interest in the field of antifungal research (Romoli et al., 2022; Zahra et al., 2022). Chinese researchers have reported that the volatile oil of Curcumae Rhizoma has a strong antifungal effect with a minimum inhibitory concentration (Wang, 2015). The prepared luliconazole cream, which includes Curcumae Rhizoma oil, is an effective antifungal of infection caused by dermatophytes namely, *Candida albicans* and *Trichophyton* spp. This antifungal effect may be credited to Curcumae Rhizoma oil in the cream formulation which enhances drug delivery into the skin and may also have a synergistic effect with luliconazole to treat athlete’s foot and ringworm (Singh et al., 2021).

*In vitro* experiments have shown that curcumol, which is the main ingredient in the volatile oil of Curcumae Rhizoma, has strong antifungal activity (Yang et al., 2007). In the clinic, ZTO vaginal suppositories are effective in the treatment of vulvovaginal and vaginal candidiasis (VVC) (Cao, 2016) and recurrent vulvovaginal and vaginal candidiasis (RVVC), with a low recurrence rate. After treatment, the symptoms, including pubic pruritus, leucorrhea, and a peculiar smell, were significantly improved (Shang, 2017). This treatment can also be administrated safely in pregnant women with fungal vaginitis (Lin et al., 2021). *In vitro* studies into the anti-candida activity of ZTO found that the mechanism of action may be through the destruction of fungal cell membrane and mitochondria (Wei and Luo, 2005). Moreover, the mechanism may also be related to repressing functions of ergosterol synthesis, mitochondrial ATPase, malate dehydrogenase, and succinate dehydrogenase activities, as well as downregulation of the relative expression of mycotoxin genes in the biosynthetic pathway. Thus, ZTO is an environmentally friendly antifungal agent with great potential (Hu et al., 2017).

### 4.4 Other infectious diseases

In addition to the abovementioned effects, Curcumae Rhizoma and its active constituents also have applications in other diseases. Curcumin has a protective effect on myocardial injury induced by Coxsackievirus B group 3 (CVB3) in mice with viral myocarditis, which may improve CVB3 virus-induced serum levels of TNF-α, IL-1β, IL-6, CK-MB, and LDH in mice by inhibiting the protein expression of caspase-3, NF-κB, and iNOS (Lu et al., 2021). Furthermore, a meta-analysis on the efficacy and safety of ZTO injection showed that the ZTO injection group had significantly higher efficiency in the treatment of hand, foot and mouth, herpetic angina and other diseases (Ma et al., 2019). In addition, Wang reported that ZTO had a strong inhibitory effect on rotavirus, it was found that the short-term efficacy of ZTO injection in the treatment of rotavirus enteritis was superior to ribavirin, which inhibits the virus and also activate blood circulation to remove blood stasis, eliminate accumulation and relieve pain, aroma and appetite, thereby improving microcirculation, promoting the regeneration of damaged epithelial cells, and promoting intestinal absorption of water and electrolyte (Wang P. L. et al., 2019).

## 5 Conclusion

The treatment of infectious diseases, especially those caused by microorganisms, has long been an issue of concern. Long-term use of antimicrobial agents leads to various adverse reactions and drug resistance. Therefore, novel drug candidates for a therapeutic agent are urgently needed. Botanical drugs and their active components have few adverse drug reactions, making them are ideal libraries for antimicrobial drug screening.

Recently, Curcumae Rhizoma has received increased attention due to its multiple therapeutic benefits against various biological targets. The main active components in Curcumae Rhizoma are non-volatile curcuminoids (curcumin) and ZTO, which act to inhibit bacterial, viral, and fungal growth. Based on our summary of the anti-microbial mechanisms of Curcumae Rhizoma, the therapeutic mechanisms of Curcumae Rhizoma may be achieved through inhibition of viral NP protein, regulation of the PI3K/Akt/mTOR signaling pathway, direct inactivation of the virus, inhibition of relevant inflammatory factors, and anti-pulmonary fibrosis. However, the mechanism responsible for microbial infection is very complex and requires further study. Studies have confirmed that a reduction in inflammatory responses suppresses viral replication and infection, inhibits concurrent bacterial infection, improves immunity, and alleviates the degree of lung function injury, with good efficacy and good safety margins, making this an ideal treatment for infected patients. Moreover, this paper highlight*s in vitro* studies of Curcumae Rhizoma to verify whether it has the anti-SARS-COV-2 effect. However, these investigations are still limited and/or have yet to be proven *in vivo*. If the anti-SARS-COV-2 effect is significant, the above speculation on the mechanism of treating COVID-19 could be further verified.

In conclusion, we hypothesize that Curcumae Rhizoma will attract increased global research interest as a potentially potent drug for the treatment of infectious diseases, as well as for overcoming the associated challenges.
